# Left ventricular outflow tract obstruction caused by abnormal mitral valve tendinous Chordae manifesting in the extremely remote period after surgery for partial atrioventricular septal defect: a case report

**DOI:** 10.1093/ehjcr/ytaf539

**Published:** 2025-10-22

**Authors:** Takanori Kawamoto, Tomohito Kogure, Takeshi Shinkawa, Kyomi Ashihara, Junichi Yamaguchi

**Affiliations:** Department of Cardiology, Tokyo Women’s Medical University, Tokyo 162-8666, Japan; Department of Cardiology, Tokyo Women’s Medical University, Tokyo 162-8666, Japan; Department of Cardiovascular Surgery, Tokyo Women’s Medical University, Tokyo 162-8666, Japan; Department of Cardiology, Tokyo Women’s Medical University, Tokyo 162-8666, Japan; Department of Cardiology, Tokyo Women’s Medical University, Tokyo 162-8666, Japan

**Keywords:** Case report, Congenital heart disease, Left ventricular outflow tract obstruction, Abnormal mitral valve chordal tendneae, Atrioventricular septal defect

## Abstract

**Background:**

Atrioventricular septal defect (AVSD) is a congenital heart disease characterized by intracardiac shunting due to septal defects, often associated with left ventricular outflow tract obstruction (LVOTO) caused by myocardial anomalies. Surgical intervention is recommended for AVSD if needed. This case report presents a rare case of LVOTO developing in the extremely remote period after partial Atrioventricular septal defect (pAVSD) surgery.

**Case Summary:**

A 35-year-old man with a history of intracardiac repair for pAVSD presented with worsening exertional dyspnoea. Transoesophageal echocardiography led to the diagnosis of LVOTO caused by systolic anterior motion (SAM) of an abnormal chordae without myocardial hypertrophy and mitral valve prolapse. Medical therapy was attempted but proved completely ineffective, so surgical resection of the abnormal chordae causing SAM was performed via open-heart surgery. Postoperatively, the LVOTO resolved, and the patient’s symptoms disappeared.

**Discussion:**

AVSD has anatomical characteristics that allow it to be associated with LVOTO in various forms; however, much remains unknown about the modes of onset and the progression of the condition. It is reported that most cases of LVOTO after intracardiac repair of AVSD occur within five years postoperatively; however, rare cases can present in the late postoperative period. Among these are cases in which symptoms become apparent due to rare anatomical features such as chordal SAM, or haemodynamic changes associated with lifestyle modifications.

Learning points

- Although rare, left ventricular outflow tract obstruction can also occur in the long-term postoperative period in case of atrioventricular septal defect.
- Chordal systolic anterior motion–induced left ventricular outflow tract obstruction is rare in atrioventricular septal defect but an important differential diagnosis.
- In adults with LVOTO, symptoms may become apparent due to lifestyle changes, even when previously minimal or absent.

## Introduction

Atrioventricular septal defect (AVSD) is a congenital heart disease characterized by intracardiac shunting due to septal defects. It often presents concomitant atrioventricular valve insufficiency and left ventricular outflow tract obstruction (LVOTO).^1^ The left ventricular outflow tract (LVOT) in the AVSD has an intrinsic predilection to develop an obstruction even if it had been repaired.^2^ Less than 5% of cases require surgical intervention for LVOTO following intracardiac repair of AVSD, and it is reported to tend to occur within 5 years after the repair.^3^ However, the factors contributing to LVOTO are not consistent.

## Summary figure

**Table:** 

Timeline	Event
At the age of 3, May	Intracardiac repair was performed following a diagnosis of partial atrioventricular septal defect.
At the age of 31, September	He had become more active in his daily life since the birth of his daughter. He began attending the gym regularly and engaging in strength training exercises.
At the age of 35, January	He began experiencing shortness of breath on exertion.
At the age of 35, April	He visited our hospital because he began experiencing shortness of breath with less exertion than before. Transthoracic echocardiography showed a high-echoic linear structure abutting the interventricular septum during systole and accelerated blood flow in the left ventricular outflow tract. Transoesophageal echocardiogram showed an abnormal chordae tendineae which caused systolic anterior motion and resulted in left ventricular outflow obstruction.
At the age of 35, December	As beta-blockers administered for symptom relief proved ineffective, surgical resection of abnormal chordae was performed.
At the age of 36, January	He became asymptomatic, and transthoracic echocardiography revealed neither abnormal chordae tendineae nor signs of left ventricular outflow tract obstruction.
At the age of 37, January	There were no findings suggestive of left ventricular outflow tract obstruction recurrence and he had been asymptomatic and was carrying out his daily activities without limitation.

## Case presentation

**Figure 1:**
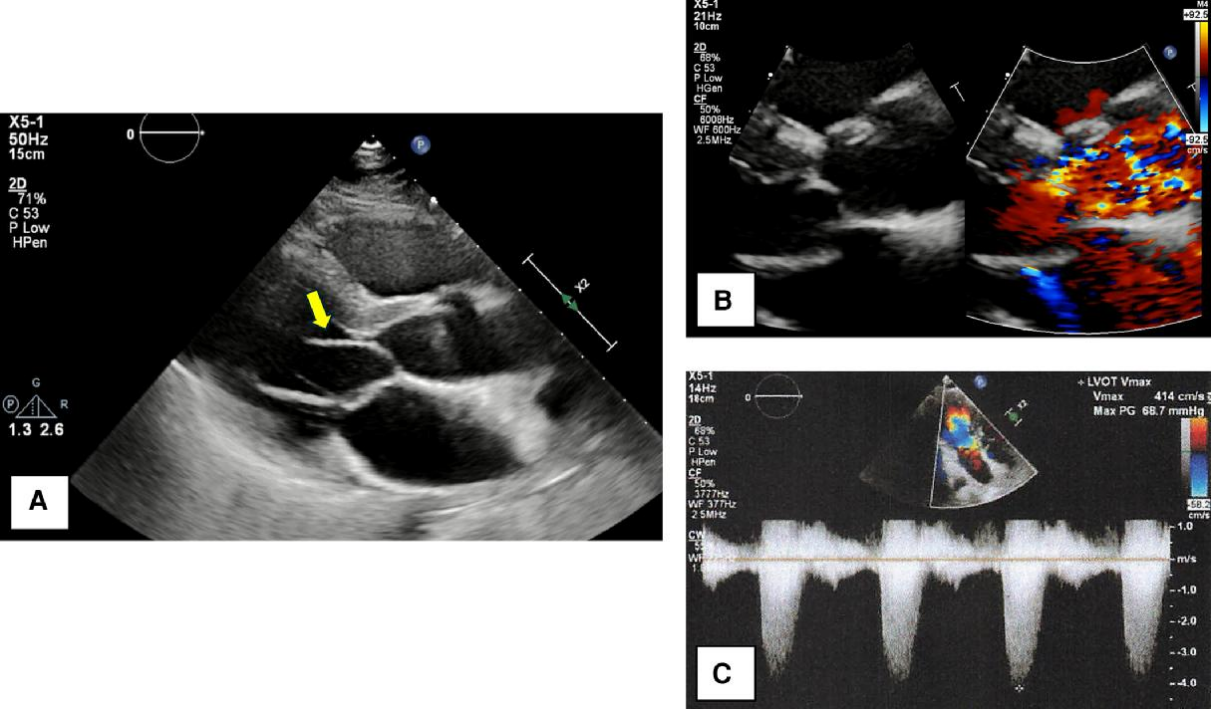
Transthoracic echocardiogram findings. (*A*) The high-echoic linear structure abutting the interventricular septum during systole was observed in long axis view of left ventricle (yellow arrow). (*B*) Zoomed image around the aortic valve in the left ventricular long-axis view. The accelerated blood flow in the LVOT with mosaic coloured Doppler was observed. (*C*) Pulse-wave Doppler image showing blood flow in the left ventricular outflow tract. The maximum flow velocity was 4.1 m/s.

**Figure 2:**
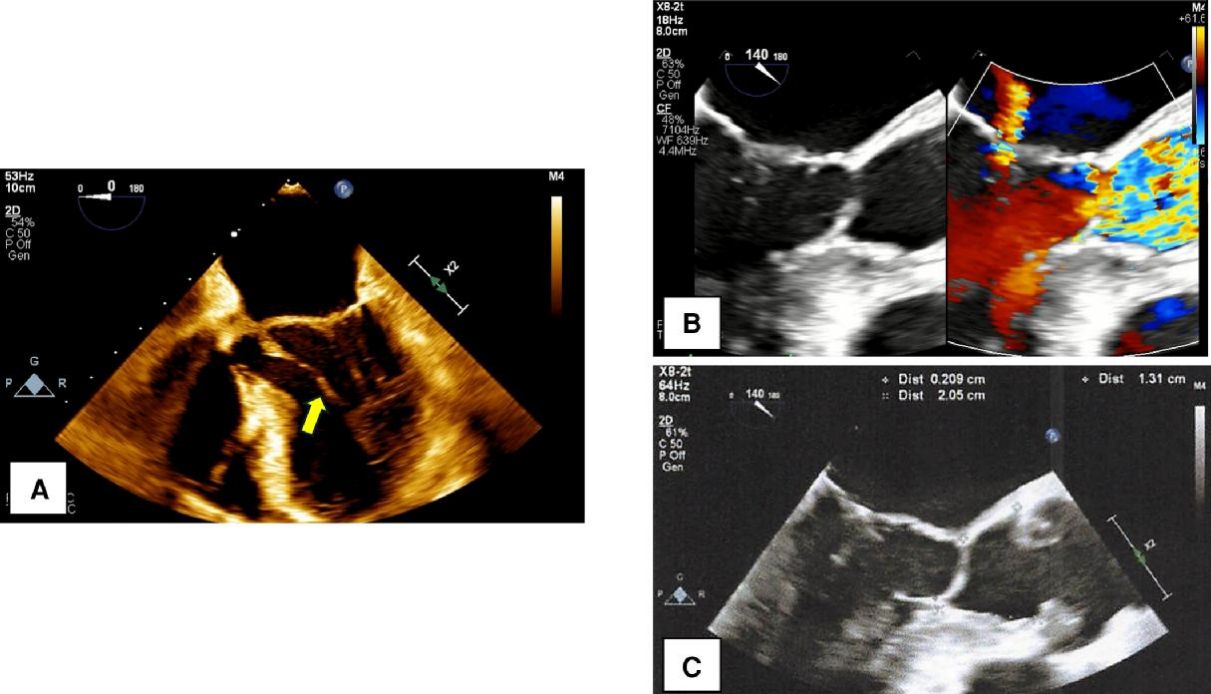
Transoesophageal echocardiogram findings. (*A*) Sepia-toned four chamber view. Abnormal chordae were observed attaching to the basal anterior mitral leaflet and the posterior papillary muscle. (*B*, *C*) Zoomed image in left ventricle outflow tract of left ventricle and aorta long axis view. The accelerated blood flow in the LVOT with mosaic coloured Doppler was observed. The narrowest point was approximately 20 mm below the aortic valve.

**Figure 3:**
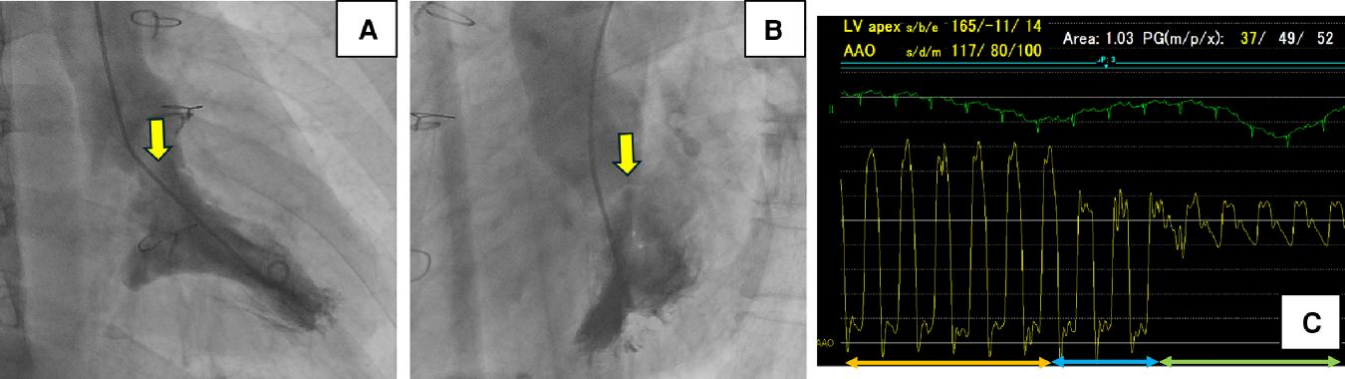
Findings related to cardiac catheterization. (*A*, *B*) Left ventriculography image during systole. The abnormal chordae tendineae were observed to interfere with the LVOT in a slit-like appearance (yellow arrow). (*C*) Pressure monitoring image during pullback from the left ventricle to the aorta. The orange arrow indicates the left ventricular apex, the blue arrow the subaortic region within the LV, and the green arrow the aortic pressure.

**Figure 4:**
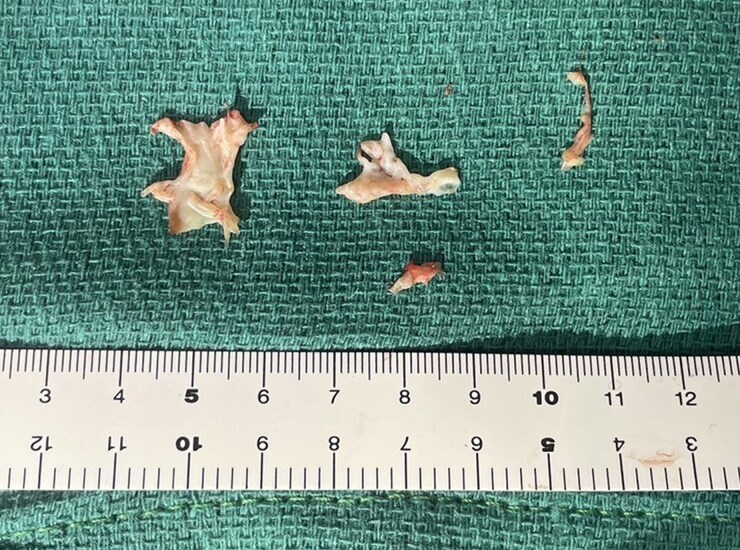
The resected abnormal chordae tendineae showed a broad, membranous structure at the site of attachment to the anterior mitral leaflet (left side of the image: mitral valve attachment; right side: papillary muscle attachment).

**Figure 5:**
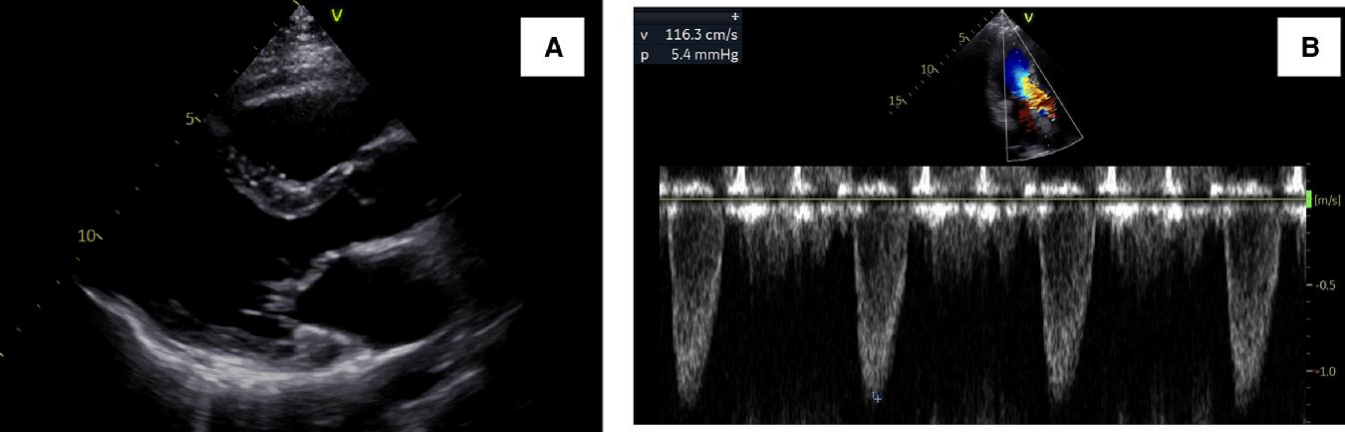
Postoperative transthoracic echocardiogram findings. (*A*) The high-echoic linear structure had vanished in long axis view of left ventricle. (*B*) Pulse-wave Doppler image showing blood flow in the left ventricular outflow tract. The maximum flow velocity was 1.16 m/s.

A 35-year-old man diagnosed with partial AVSD (pAVSD) presented to our hospital with progressive exertional dyspnoea for the past 3 months. After the birth of his daughter four years ago, he began regularly engaging in resistance training at the gym. He underwent intracardiac repair (single-patch method of atrial septal defect closure using Gore-Tex® 8 ply and mitral valve repair with suturing cleft) at the age of 3 for pAVSD with concomitant mitral regurgitation. He remained asymptomatic until presenting to our hospital after surgery. On admission he exhibited systolic heart murmur at the second intercostal space on the right sternal border. Breath sounds were clear, and no oedema was noted throughout the body. The electrocardiogram showed sinus rhythm with prolonged PQ interval, left axis deviation, and signs of left ventricular hypertrophy without ST changes, and a heart rate of 75 beats per minute. Chest radiography showed no cardiomegaly and no abnormalities in the lung fields. Laboratory test results showed a normal range of blood count, liver enzymes, creatinine level, and slightly elevated brain natriuretic peptide level (25.0 pg/mL). Transthoracic echocardiography (TTE) showed a high-echoic linear structure abutting the interventricular septum during systole and accelerated blood flow in the LVOT with mosaic coloured Doppler (*Figure 1A, B* and Supplementary material online, *Video S1*, *S2*), maximum flow velocity was 4.1 m/s (*Figure 1 C*) and systolic aortic-left ventricular pressure gradient was 64 mmHg, without accompanying myocardial wall thickening (8 mm of inter ventricular septal thickness and posterior wall thickness) or systolic anterior motion (SAM) of the mitral valve. The left ventricular diameter and wall motion were normal (46 mm of left ventricular end diastolic diameter, 112 mL of left ventricular end diastolic volume), no residual intra-cardiac shunt and no valve dysfunction requiring intervention was observed. No collapse of the inferior vena cava was observed. Transoesophageal echocardiogram (TEE) showed an abnormal chordae tendineae attached to the annular portion of the anterior mitral leaflet and posterior papillary muscle, which caused SAM and resulted in LVOTO (*Figure 2A, B* and Supplementary material online, *Video S3*). The narrowest part was located 21 mm below the aortic valve, and the minimum diameter of the left ventricular outflow tract due to chordal SAM was 2 mm (*Figure 2C*). Intra-cardiac pressures including right atrium, right ventricle, pulmonary artery and pulmonary capillary wedge pressure were normal by right heart catheter. Coronary angiography revealed intact coronary artery. Left ventriculography did not reveal obvious LVOT narrowing, but the abnormal chordae tendineae were observed to interfere with the LVOT in a slit-like appearance (*Figure 3A, B*). The Brockenbrough phenomenon was observed as a > 10 mmHg decrease in systolic arterial pressure after ventricular premature contractions during catheterization, and the systolic pressure gradient between the left ventricular apex and the left ventricular outflow tract was 49 mmHg (*Figure 3C*). Contrast-enhanced computed tomography showed no calcification in the heart, including the pericardium, but showed a short distance between the sternum and the heart, suggesting adhesion. Based on all findings, the cause of exertional dyspnoea was diagnosed as LVOTO. To relieve symptoms, oral bisoprolol 1.25 mg once daily was initiated, however there was no improvement in symptom and findings of LVOTO in TTE at all, leading to the decision to proceed with open-heart surgery. Open-heart surgery was performed under general anaesthesia and TEE guidance, and abnormal chordae tendineae resection was performed. Intraoperative findings showed a normal aortic valve with no abnormal structures attached to the subaortic lesion. A redundant abnormal chordae tendineae was observed from the aortic side, interfering with the LVOT. It extended from the posterior papillary muscle to the basal anterior mitral leaflet, forming a membranous structure. (*Figure 4*). After resection, the LVOT blood flow velocity decreased to 1.16 m/s, and the mosaic coloured Doppler disappeared without progression of mitral regurgitation in the TTE (*Figure 5*, Supplementary material online, *Video S4*). Postoperatively, exertional dyspnoea resolved, and no recurrence of LVOTO has been observed after one year of follow-up. Given the diverse mechanisms of LVOTO in AVSD, ongoing follow-up in the outpatient setting with TTE is scheduled.

## Discussion

Surgical outcomes for pAVSD are generally favourable; however, there remains a certain risk of reoperation.^4^ LVOTS is the second most common reason for reoperation following pAVSD repair, and its causes are diverse.^5^ The most common cause of LVOTO is subaortic fibroelastic membrane. Other reported causes include septal hypertrophy, atrioventricular valve, and chordal or papillary muscle abnormalities.^6^ Piccoli *et al*. noted that papillary muscle malposition and left ventricular hypoplasia are key anatomical contributors in AVSD cases.^7^ When the atrioventricular valve lies close to the ‘scooped out’ interventricular septum, the valve and chordae tendineae often lie in close proximity to the LVOT, making the occurrence of LVOTO more likely even if after repair of AVSD. In this adult patient, no evidence of left ventricular hypoplasia was observed, and both echocardiographic and intraoperative assessments showed normal papillary muscle positioning. Meanwhile, despite the absence of a ventricular communication, pAVSD shares the characteristic anatomic features of a ‘scooped-out’ ventricular septum and an elongated left ventricular outflow tract.^8^ What is particularly interesting in this case is the development of LVOTO and the progression of symptoms in the long-term postoperatively, as well as the underlying cause of the LVOTO. The incidence of LVOTO over a 30-year period following intracardiac repair of pAVSD is reported to be only 4.4%, mostly within the first five years postoperatively. It is more common in cases requiring surgical intervention.^3^ Furthermore, Chordal SAM is a rare endocardial change.^9^ This case is notable for the unusually late onset of LVOTO after pAVSD repair and the rare occurrence of chordal SAM without left ventricular hypertrophy. At the initial intracardiac repair, no LVOTO was observed and only atriotomy was performed, likely leaving abnormal chordae tendineae undetected. Given the clinical course, increased cardiac output associated with physical growth and the initiation of regular resistance training^10,11^ might have induced chordal SAM via the Venturi effect, resulting in LVOTO. It was considered that the patient’s symptoms might have been masked during the pre-symptomatic period due to the circumstances of the COVID-19 pandemic, during which he was working remotely. Additionally, as this was prior to the birth of his child, his overall activity level was likely low, further contributing to the absence of overt symptoms. Although subaortic stenosis in adults typically progresses slowly,^12^ these lifestyle changes may have precipitated the subacute onset of symptoms. The surgical outcomes for LVOTO associated with AVSD are generally favourable; however, the recurrence rate tends to increase over time following initial intervention. As in the present case, there are rare instances where LVOTO is detected in the late postoperative period after complete repair of pAVSD, highlighting the importance of continuous follow-up.

## Conclusion

AVSD has anatomical characteristics that allow it to be associated with LVOTO in various forms; however, much remains unknown about the modes of onset and the progression of the condition. Therefore, long-term follow-up and careful monitoring are essential.

## Lead author biography


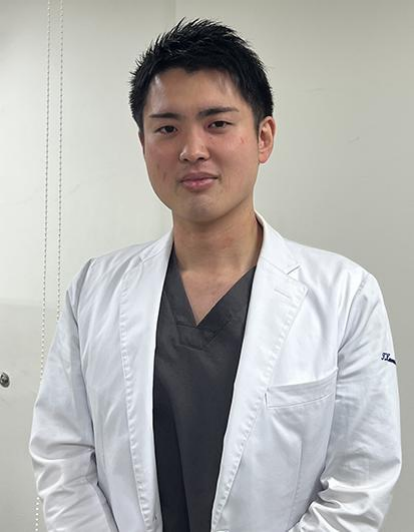


A cardiologist specializing in cardiovascular intervention, structural heart disease, and echocardiography. Actively involved in the care of congenital heart disease patients by applying expertise in these specialized fields.

## Supplementary Material

ytaf539_Supplementary_Data

## References

[1] Manning PB . Partial atrioventricular canal: pitfalls in technique. Semin Thorac Cardiovasc Surg Pediatr Card Surg Ann 2007;10:42–46. 10.1053/j.pcsu.2007.02.002 17433990

[2] Perez Y, Dearani AJ, Miranda RW, Stephens EH. Subaortic stenosis in adult patients with atrioventricular septal defect. Ann Thorac Surg 2023;115:479–484. 35987344 10.1016/j.athoracsur.2022.08.011

[3] Ivanov Y, Buratto E, Naimo P, Lui A, Hu T, Udekem Y, et al Incidence and management of the left ventricular outflow obstruction in patients with atrioventricular septal defects. Interactive Cardiovasc Thorac Surg 2022;34:604–610. 10.1093/icvts/ivab303 PMC8972236 34751750

[4] Devlin PJ, Backer CL, Eltayeb O, Monge MC, Hauck AL, Costello JM. Repair of partial atrioventricular septal defect: age and outcomes. Ann Thorac Surg 2016;102:170–177. 27112649 10.1016/j.athoracsur.2016.01.085

[5] Stulak JM, Burkhart HM, Dearani JA, Cetta F, Barnes RD, Connolly HM, et al Reoperations after repair of partial atrioventricular septal defect: a 45-year single-center experience. Ann Thorac Surg 2010;89:1352–1359. 20417744 10.1016/j.athoracsur.2010.01.018

[6] Gallo P, Formingari R, Hokaye NJ, Offizi FD, Alessandro D, Francalani P, et al Left ventricular outflow tract obstruction in atrioventricular septal defects: a pathologic and morphometric evaluation. Clin Cardiol 1991;14:513–521. 1810690 10.1002/clc.4960140611

[7] Piccoli GP, Ho SY, Wilkinson JL, Macartney FJ, Gerlis LM, Anderson RH, et al Left-sided obstructive lesions in atrioventricular septal defects: an anatomic study. J Thorac Cardiovasc Surg 1982;83:453–460. 7062757

[8] Overman DM . Reoperation for left ventricular outflow tract obstruction after repair of atrioventricular septal. Semin Thorac Cardiovasc Surg Pediatr Card Surg Ann 2007;10:42–46. 10.1053/j.pcsu.2014.01.008 24725716

[9] Pearson AC, Pasierski TJ, Orsinelli DA. Systolic anterior motion of the mitral chordae tendineae: prevalence and clinical and Doppler-echocardiographic features. Am Heart J 1996;131:748–753. 8721650 10.1016/s0002-8703(96)90282-3

[10] Hambrecht R, Gielen S, Linke A, Fiehn E, Yu J, Walther C, et al Effects of exercise training on left ventricular function and peripheral resistance in patients with chronic heart failure; a randomized trial. JAMA 2000;283:3095–3101. 10865304 10.1001/jama.283.23.3095

[11] Lassing J, Maudrich T, Kenville R, Uyar Z, Biscoff C, Fikenzer S, et al Intensity–dependent cardiopulmonary response during and after strength training. Sci Rep 2023;13:6632. 37095279 10.1038/s41598-023-33873-x PMC10126007

[12] Oliver JM, Gonzalez A, Gallego P, Recalde AS, Benito F, Mesa JM. Discrete subaortic stenosis in adults: increased prevalence and slow rate of progression of the obstruction and aortic regurgitation. J Am Coll Cardiol 2001;38:835–842. 11527642 10.1016/s0735-1097(01)01464-4

